# Immunohistochemical detection of *Mycoplasma salivarium* in oral lichen planus tissue

**DOI:** 10.1111/jop.12568

**Published:** 2017-04-06

**Authors:** Harumi Mizuki, Ryosuke Abe, Shintaro Kogi, Toshinari Mikami

**Affiliations:** ^1^ Division of Oral and Maxillofacial Surgery Department of Oral and Maxillofacial Reconstructive Surgery School of Dentistry Iwate Medical University Morioka Iwate Japan; ^2^ Division of Anatomical and Cellular Pathology Department of Pathology Iwate Medical University Morioka Iwate Japan

**Keywords:** etiology, immunohistochemistry, *Mycoplasma salivarium*, oral lichen planus, sawtooth rete ridge

## Abstract

**Background:**

Oral lichen planus (OLP) is a T‐cell‐mediated inflammatory disease; however, its exact etiology is unknown. Hyperkeratosis is often observed in OLP lesions. Previous studies have revealed the localization of *Mycoplasma salivarium* in the epithelial cells of oral leukoplakia with hyperkeratosis. Herein, we investigated the presence of *M. salivarium* in OLP tissue by immunohistochemistry to determine the causative factor of OLP.

**Methods:**

Forty‐one formalin‐fixed, paraffin‐embedded samples obtained from 31 patients with OLP were examined. Ten samples of normal‐appearing oral mucosa were used as controls. Immunohistochemistry (IHC) was performed using anti‐*M. salivarium* monoclonal antibodies.

**Results and Conclusions:**

*Mycoplasma salivarium* was detected in the epithelium and lymphocyte infiltrate area in 24 of 41 OLP samples (58.5%). The bacteria were intracellularly localized in epithelial cells, while it was unclear whether they were also localized in lymphocyte cells or in the extracellular spaces among the lymphocytes in the subepithelial lymphocyte infiltrate area. Little or no staining was observed in the epithelium in the normal‐appearing mucosa samples. Sawtooth rete ridge formation was observed in 21 OLP samples (51.2%), and a significant positive correlation between sawtooth rete ridge formation and IHC positivity was demonstrated. However, the role of *M. salivarium* in the epithelium and lamina propria of OLP tissue remains unknown.

## 1 INTRODUCTION

Oral lichen planus (OLP) is a chronic mucocutaneous inflammatory disease with an unknown etiology. It presents alone or with concomitant skin lesions and commonly affects the buccal mucosa, tongue, and gingiva. OLP is predominantly found in the buccal mucosa and is often found in multiple sites.^1^

The clinical features of OLP are commonly classified according to six patterns, including reticular, erosive, atrophic, plaque‐like, papule, and bullous patterns.^2^ These features often coexist in the same patient, and the most common among these clinical forms is the reticular type with a lace‐like pattern and whitish lines within the papules, referred to as “Wickham striae”.^3^

The essential histopathological features of OLP include liquefaction degeneration of the basal cells of the epithelium, a band‐like lymphocytic infiltrate at the epithelial‐stromal junction with obfuscation of the basal cell region, and normal epithelial maturation.^4^, ^5^ Additionally, hyperkeratosis, parakeratosis, a sawtooth rete ridge, and civatte bodies are features of OLP.^6^

Oral lichen planus is considered a T‐cell‐mediated inflammatory disease, and the infiltrating lymphocytes in the subepithelial region mainly consist of CD4+ and CD8+ T cells.^7^ Liquefaction degeneration of the basal cells is induced by apoptosis, which is primarily triggered by CD8+ T cells.^8^ However, its precise mechanism is unclear, and the antigen that triggers the inflammatory response of the T cells remains unknown.

Several causative factors of OLP have been proposed, including infectious viruses and bacteria, dental materials, drugs, and autoimmunity.^6^ With regard to dental materials and drugs, the presence of oral mucosal lesions has been reported, which are improved or eliminated by removing the dental restorative materials or drug withdrawal.^9^ It is difficult to distinguish these lesions from OLP because they are clinically and histopathologically similar to OLP. However, these lesions are regarded as different than OLP and are referred to as “oral lichenoid lesions (OLLs)”.^9^

The association of various infectious agents, such as viruses and bacteria, with OLP has been studied. Some viruses, including human papilloma virus (HPV) and hepatitis C virus (HCV), have been detected at higher rates in patients with OLP than in control subjects. However, the relationship between infection with these viruses and OLP is controversial.^10^ Plaque control can improve the symptoms of OLP, suggesting an association of some oral bacteria with OLP.^11^ Recently, several pathogenic periodontal bacteria were detected at higher levels in patients with OLP than in non‐OLP patients.^12^ However, the correlation between these bacteria and OLP is also unclear.

Recently, Choi et al.^13^ reported the presence of bacteria throughout the epithelium and lamina propria of OLP tissues, and they proposed that intracellular bacteria trigger T‐cell infiltration and provide target antigens. However, the species of bacteria detected in the OLP tissues was not clarified.

Mycoplasmas are the smallest free‐living bacteria capable of self‐replication. Among the 16 mycoplasmas isolated from humans, *M. salivarium* is the most common species isolated from the oral cavity.^14^ It preferentially resides in dental plaques and gingival sulci, similar to pathogenic periodontal bacteria.^15^

Mizuki et al.^16^ demonstrated an intracellular localization of *M. salivarium* in the epithelial cells of oral leukoplakia (OL). Although the role of *M. salivarium* in OL is unclear, a close relationship between the localization of *M. salivarium* in epithelial cells and hyperkeratosis of the epithelium of the oral mucosa has been suggested.

Hyperkeratosis is regarded as a histopathological feature of OLP. Wickham striae appear in reticular types of OLP, corresponding to the focal regions of hyperkeratosis or parakeratosis.^17^ Therefore, it has been postulated that *M. salivarium* cells are present in the epithelia of OLP tissues, particularly in those with reticular OLP. However, no report has demonstrated the presence of mycoplasmas, including *M. salivarium*, in OLP lesions.

In this study, we detected *M. salivarium* in OLP tissues by immunohistochemistry (IHC) using anti‐*M. salivarium* monoclonal antibodies to investigate the causative factor of OLP.

## 2 MATERIALS AND METHODS

### 2.1 Materials for IHC

This study was approved by the Human Investigation Committee (No. 1202) of our institution (Table 1).

**Table 1:** Clinical and histopathological features of immunohistochemistry‐positive OLP samples

No.	Age	Sex	Clinical features		Location of biopsy	Histopathological features			
			Location	Type		Hyper‐/Para‐keratosis	Atrophy	Sawtooth rete ridge	Civatte body
1	76	F	Cheek (unilateral)	Reticular	Gingiva	**+**		**+**	
			Gingiva (unilateral)						
2	73	F	Cheek (bilateral)	Reticular	Cheek	**+**		**+**	**±**
			Gingiva (unilateral)						
3	50	M	Cheek (bilateral)	Reticular	Cheek	**+**		**+**	
			Tongue (unilateral)	Erosive	Tongue	**+**		**+**	**±**
4	71	F	Cheek (bilateral)	Reticular	Cheek	**+**		**+**	
5	78	F	Cheek (bilateral)	Reticular	Cheek	**+**	**+**	**+**	**±**
			Gingiva (bilateral)	Reticular					
6	65	F	Cheek (bilateral)	Erosive	Cheek	**+**		**+**	
			Gingiva (unilateral)	Erosive					
7	66	F	Tongue (unilateral)	Atrophic	Tongue	**+**		**+**	
			Cheek (unilateral)	Reticular					
8	50	F	Cheek (unilateral)	Reticular	Cheek	**+**		**+**	
9	53	M	Cheek (bilateral)	Reticular	Cheek (right)	**+**		**+**	
				Reticular	Cheek (left)	**+**	**+**	**+**	
10	82	M	Gingiva (unilateral)	Atrophic	Gingiva	**+**		**+**	
11	67	F	Cheek (unilateral)	Reticular	Cheek	**+**		**+**	**+**
12	77	F	Cheek (unilateral)	Reticular	Cheek	**−**		**+**	
			Gingiva (bilateral)	Atrophic	Gingiva	**+**	**+**	**+**	
13	61	F	Tongue (unilateral)	Atrophic	Tongue	**+**	**+**	**+**	
14	53	F	Cheek (bilateral)	Reticular	Cheek	**+**		**+**	
			Tongue (bilateral)	Reticular					
15	35	M	Cheek (bilateral)	Reticular	Cheek	**+**		**+**	
16	61	F	Cheek (unilateral)	Reticular	Cheek	**+**			
17	73	M	Gingiva (unilateral)	Reticular	Gingiva	**+**	**+**		
			Cheek (unilateral)	Atrophic	Cheek	**+**	**+**		
18	76	F	Cheek (bilateral)	Reticular	Cheek (right)	**+**			**+**
			Gingiva (bilateral)	Reticular	Cheek (left)	**+**			**+**
19	38	F	Cheek (unilateral)	Reticular	Cheek	**+**	**+**		**+**

No. 19: A sample showed positive staining only in the subepithelial region in this case.

Formalin‐fixed and paraffin‐embedded (FFPE) samples of OLP tissues were used for hematoxylin and eosin staining and IHC. Biopsied tissue specimens were obtained from patients with oral mucosal lesions showing reticular, erosive, or atrophic changes who were examined at the Division of Oral and Maxillofacial Surgery (Department of Oral and Maxillofacial Reconstructive Surgery, School of Dentistry, Iwate Medical University).

Ten FFPE samples containing normal‐appearing oral mucosa, which were obtained by excising cysts, benign tumors, or other diseases, were used as controls. The mean age of the 10 patients (four males and six females) was 59 years (range: 31‐79 years).

Specimens were cut into 4‐μm‐thick serial sections and mounted onto MAS‐coated slides (Matsunami, Osaka, Japan). The slides were deparaffinized, rehydrated, and used for hematoxylin and eosin staining and IHC.

### 2.2 Histopathological diagnosis of OLP

Histopathological diagnosis was performed using slides stained with hematoxylin and eosin, according to the histopathological criteria of OLP by Eisenberg.^4^

Samples with findings of liquefaction degeneration of the basal cells, a subepithelial dense band‐like lymphocytic infiltrate, and a normal epithelial maturation pattern, which are essential features for the histological diagnostic criteria of OLP, were diagnosed as OLP.

After the histological diagnosis, 41 samples obtained from 31 patients were selected for further analysis.

The mean age of the 31 patients (seven males and 24 females) was 62 years (range: 35‐82 years). The locations and clinical features of OLP and the locations of the biopsies are shown in Tables 1 and 2.

**Table 2:** Clinical and histopathological features of immunohistochemistry‐negative OLP samples

No.	Age	Sex	Clinical features		Location of biopsy	Histopathological features			
			Location	Type		Hyper–/Para‐keratosis	Atrophy	Sawtooth rete ridge	Civatte body
1	62	F	Cheek (bilateral)	Reticular	Cheek	**+**	**+**		
2	39	F	Cheek (bilateral)	Reticular	Cheek	**+**		**+**	
3	50	F	Cheek (bilateral)	Reticular	Cheek (right)	**+**		**+**	
			Lip (unilateral)	Reticular	Cheek (left)	**+**			
				Reticular	Lip	**+**		**+**	
4	57	F	Cheek (bilateral)	Reticular	Cheek	**+**			
5	63	F	Cheek (bilateral)	Atrophic	Cheek (right)	**+**	**+**		
				Atrophic	Cheek (left)	**+**			
6	52	M	Cheek (bilateral)	Erosive	Cheek (right)	**+**	**+**		
			Lip (unilateral)	Erosive	Cheek (left)	**−**	**+**		
7	39	F	Tongue (unilateral)	Erosive	Tongue	**+**	**+**		
8	79	M	Cheek (unilateral)	Erosive	Tongue	**+**	**+**		
9	74	F	Tongue (bilateral)	Erosive	Cheek	**+**	**+**		
10	66	F	Cheek (bilateral)	Erosive	Cheek	**+**	**+**		**+**
11	62	F	Cheek (unilateral)	Reticular	Cheek	**+**			
			Gingiva (unilateral)	Reticular	Gingiva	**+**			
12	51	F	Cheek (bilateral)	Reticular	Cheek	**+**	**+**		

### 2.3 IHC

Immunohistochemistry staining was performed using a biotin‐free, tyramide‐catalyzed, signal amplification system (CSA II system; Dako, Carpinteria, CA, USA) with an anti‐*M. salivarium* monoclonal antibody (MAb), as described previously.^16^

## 3 RESULTS

Tables 1 and 2 show the results of IHC and the histopathological features of the OLP samples, including hyperkeratosis, parakeratosis, atrophy, sawtooth rete ridges, and civatte bodies.

**Figure 1:**
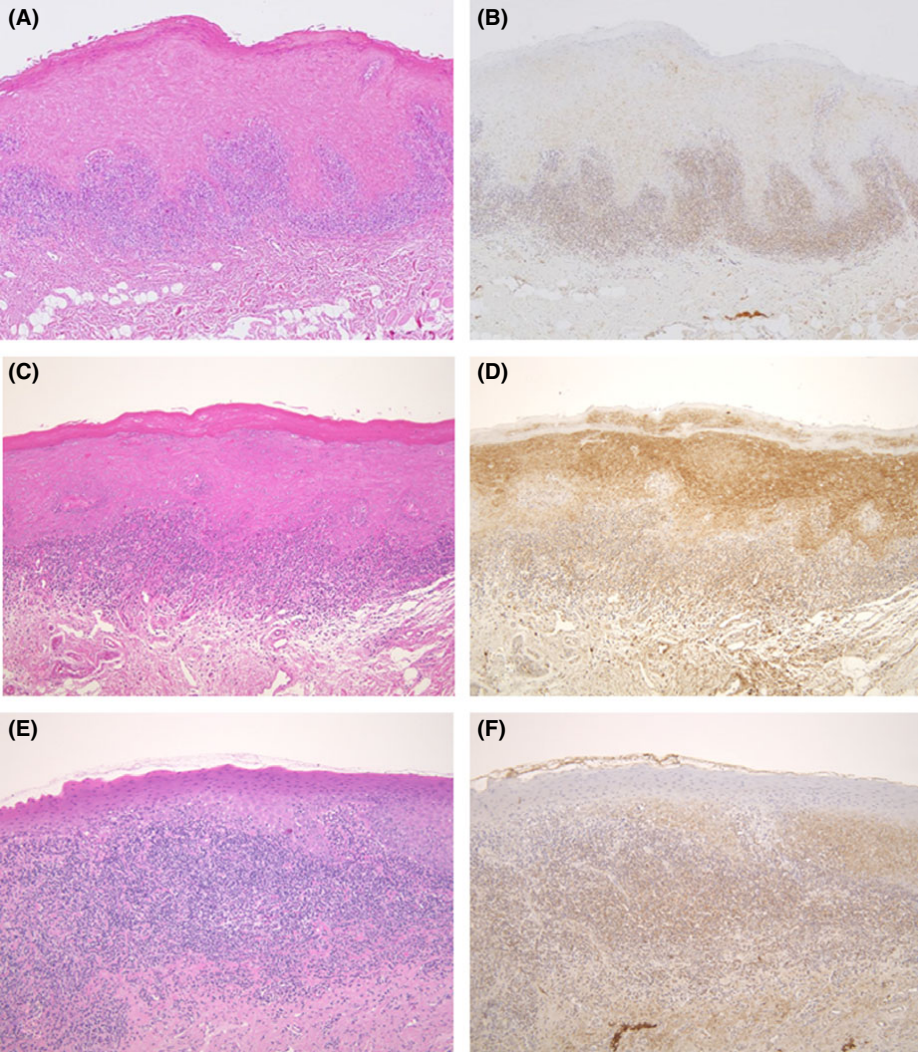
Hematoxylin and eosin (A, C, E) and immunohistochemical (B, D, F) staining of OLP samples showing positive reactions with or without the formation of a sawtooth rete ridge

All samples stained with hematoxylin and eosin showed evidence of OLP (Figures 1A,C,E, and 3A,C,E).

The IHC findings indicated that 24 of the 41 samples (58.5%) exhibited positive reactions against *M. salivarium* in the epithelium and lymphocyte infiltrate area (Figure 1B,D,F). In 23 samples, positive staining was observed throughout the epithelium and lymphocyte infiltrate area, but one showed positive staining in the lymphocyte infiltrate area, not in the epithelium. The degree of IHC staining varied among the samples and the areas within the individual samples (Figure 1B,D, F).

Various degrees of positive reactions were observed in the epithelial cells. These reactions were observed in both the upper and lower parts of the epithelium in 14 samples (Figure 1B,D), but they were mainly in the lower part of the epithelium in nine samples. Staining of the subepithelial region was mostly confined to the areas of lymphocyte infiltration (Figure 1B,D,F).

**Figure 2:**
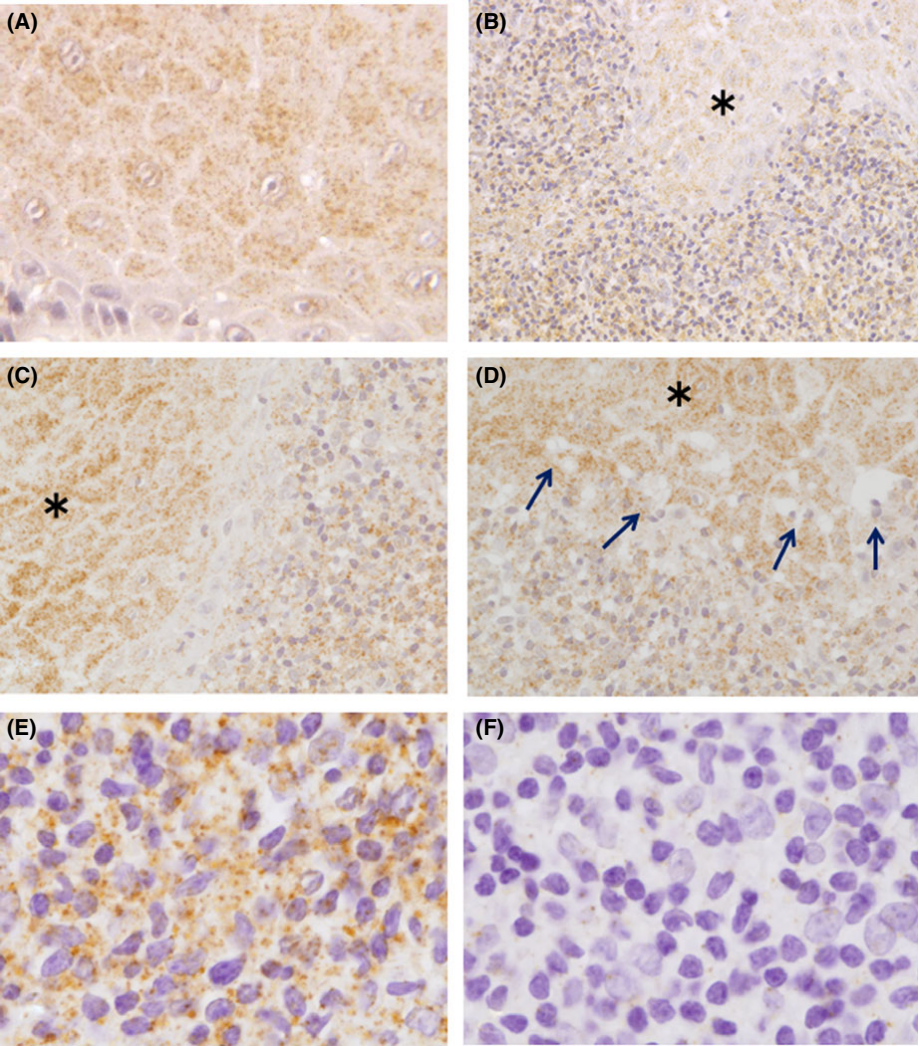
Immunohistochemical staining of OLP samples showing positive reactions. Positive staining in the epithelial cells (A), in the interface between the epithelium (*) and the lamina propria (B‐D), and in the subepithelial lymphocyte infiltration area showing positive staining (E) or no staining (F). The arrows shown in D indicate the vacuoles at the bottom of the epithelium

The interface between the epithelium and lamina propria was typically indistinct (Figure 2B‐D), and positive reactions were abundant throughout the epithelium and the lymphocyte infiltrate area (Figure 2B‐D). In some of the positive samples, vacuoles (arrows) with or without a nucleus were observed in the basal cell layers of the epithelium (Figure 2D).

Immunohistochemistry staining was observed at the lymphocyte infiltrate areas, but it was unclear whether the stains localized to the cells or the intercellular spaces (Figure 2E).

**Figure 3:**
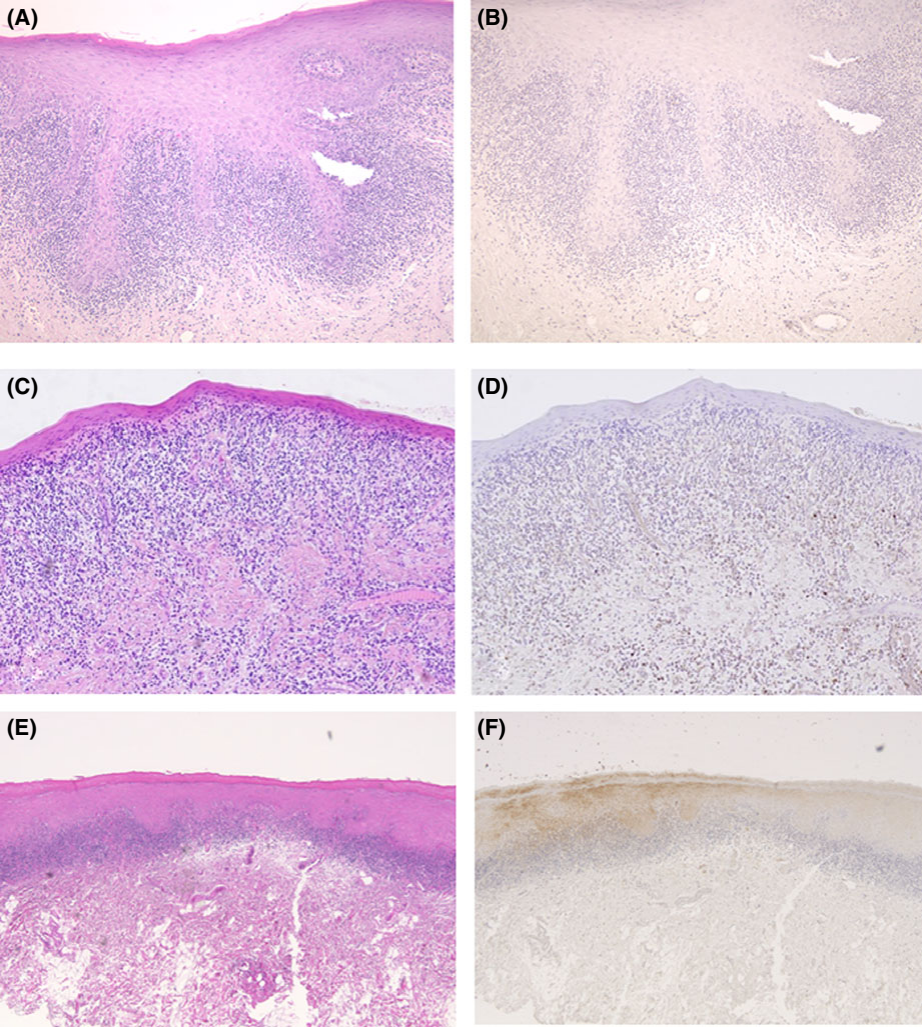
Hematoxylin and eosin staining (A, C) and immunohistochemical staining (B, D) of OLP samples showing no positive staining. Hematoxylin and eosin staining (E) and immunohistochemical staining (F) of an OLP sample showing both positive and negative immunohistochemical results

Seventeen samples (41.5%) demonstrated no positive reaction in the epithelium or the lymphocyte infiltrate area (Figure 3B,D). Specifically, no positive reaction was observed in the atrophic epithelium or in the subepithelial lymphocyte infiltrate areas under the atrophic epithelium (Figure 3D).

Some samples exhibited both positive and negative areas within the same section (Figure 3F). Little or no staining was observed in the epithelial layer, and no staining was observed in the subepithelial region in the normal‐appearing mucosa samples.

No relationship was found between the clinical OLP subtypes and IHC positivity for the anti‐*M. salivarium* MAb. As shown in Tables 1 and 2, hyperkeratosis and parakeratosis of the epithelium were observed in five (12.2%) and 34 (82.9%) samples, respectively. Atrophy (thinning) of the epithelium was seen in 16 samples (39.0%). Some samples showed both atrophy and hyperkeratosis or parakeratosis of the epithelium in a single section.

Sawtooth rete ridges were observed in 21 of 41 samples (51.2%); 18 of these 21 samples (78.5%) demonstrated positive IHC reactions in the epithelium and the lymphocyte infiltrate area (Figure 1B,D), but three of the samples were negative (Figure 3B). Conversely, 14 of the 20 samples without the sawtooth rete ridge pattern were negative in the epithelium and subepithelial region (Figure 3D). The remaining six samples exhibited positive reactions in the epithelium and lymphocyte infiltrate area or only in the lymphocyte infiltrate area. Therefore, a significant correlation was noted between the sawtooth rete ridge pattern and IHC positivity (*P*=.0004). The data were analyzed using Fisher's exact test.

Civatte bodies were observed in eight of the samples (19.5%).

## 4 DISCUSSION

OLP is a T‐cell‐mediated inflammatory disease. However, the exact antigen that triggers the inflammatory response of the T cells is unknown,^18^ although some infectious microorganisms have been proposed as an etiological factor of OLP.

Associations between OLP and viruses, including Herpes simplex 1, Epstein‐Barr virus, cytomegalovirus, Herpes virus 6, HPV, and HCV, have been studied.^10^ In particular, HPV has been detected in OLP tissues, but the detection rates have varied greatly among investigations.^10^ In contrast, many investigators have shown a significantly higher risk of HCV infection in patients with OLP than in control subjects, although geographic regional differences among the studies were found.^19^ Some studies also suggested that patients with HCV are more likely to develop OLP. However, the mechanism involved in OLP development in patients with HCV is not clear, and the localization of HCV in OLP lesions is controversial.^19^

In addition, higher levels of infection with several pathogenic periodontal microorganisms, including *Aggregatibacter actinomycetemcomitans*,* Porphyromonas gingivalis*,* Prevotella intermedia*,* Tannerella forsythia*, and *Treponema denticola*, have been reported in patients with OLP than those in non‐OLP patients.^12^ Although the role of these bacteria in OLP lesions is uncertain, an improvement of the symptoms of OLP by removal of the plaques and calculi has been reported.^20^ This finding may suggest that periodontal bacteria contribute to the inflammation associated with OLP.

We demonstrated, for the first time, the presence of *M. salivarium* in OLP tissues by IHC using anti‐*M. salivarium* monoclonal antibodies. *M. salivarium* was detected in 24 of 41 (58.5%) samples and in 19 of 31 (61.3%) cases by IHC using anti‐*M. salivarium* MAb. Furthermore, detection occurred in the epithelium and the lymphocyte infiltrate area in 24 samples. The bacteria were intracellularly localized in epithelial cells, while it was unclear whether they were also localized in lymphocyte cells or in the extracellular spaces among the lymphocytes in the subepithelial lymphocyte infiltrate area.

In this study, 12 cases with unilateral lesions were included among 31 cases. The modified WHO diagnostic criteria for OLP propose that the lesions satisfying clinical criteria, including the presence of bilateral, more or less symmetrical lesions, as well as histopathological criteria, be diagnosed as OLP and that the lesions showing histopathologically typical conditions but clinically compatible conditions of OLP be diagnosed as OLL.^5^ In accordance with these criteria, the 12 unilateral cases should have been excluded from the present study. However, these cases were included because the lesions can occur unilaterally as well as bilaterally when topical infection with microorganisms, including mycoplasma, is an etiological factor. Incidentally, the positive rate of IHC among 19 cases, excluding 12 unilateral cases, was 10 of 19 (52.6%), which was not greatly different from that (61.3%) among the 31 total cases. However, in future studies for elucidating the role of *M. salivarium* in OLP, unilateral cases should be excluded from the subjects.

Recently, Choi et al.^13^ reported that abundant bacteria were detected throughout the epithelium and the lamina propria of OLP tissues by in situ hybridization using a universal probe targeting the bacterial 16S rRNA gene. The incidence of bacterial detection in the basal layer of the epithelia was significantly different between control tissues and OLP tissues (10.0% vs 72.5%, respectively).^13^ Additionally, the levels of bacteria detected within the lamina propria exhibited a significantly positive correlation with those within the epithelia in the OLP but not in the control tissues.^13^ Although the species of bacteria detected by in situ hybridization are not clarified, their distribution in OLP tissues is highly consistent with that of *M. salivarium* detected in the epithelia and the subepithelial lymphocyte infiltrate areas. This finding may suggest that the bacteria included *M. salivarium*.

Bacteria localizing in the epithelium or lamina propria of OLP tissues are usually identified by microscopy, particularly electron microscopy. However, no previous studies observing OLP tissues using electron microscopy have revealed the presence of bacteria in OLP tissues, although some studies demonstrated the presence of membrane‐coating granules, electron‐dense granules, vacuoles, and lipid droplet‐like structures.^21^, ^22^, ^23^ These structures are also observed in oral leukoplakia tissues and are suggested to correspond to the images of mycoplasma (H. Mizuki unpublished data).

Although sawtooth rete ridge formation is recognized to be less prevalent in OLP than in the lichen planus of the skin, it was reportedly observed in 30% of OLP cases.^3^ In our study, sawtooth rete ridge patterns were found in 21 of 41 (51.2%) samples. The difference between the rate reported in the previous study and that of this study may result from differences in the criteria for defining sawtooth rete ridge alteration. Nevertheless, a significant positive correlation between sawtooth rete ridge formation and IHC positivity may suggest that *M. salivarium* does not randomly infect the epithelium and lamina propria of OLP tissues with or without sawtooth rete ridges, and sawtooth rete ridges are induced by *M. salivarium* infection in the epithelium and/or subepithelial region of the lamina propria. However, the mechanism of sawtooth rete ridge formation remains unknown.

The vacuoles were observed in the basal cell layer of the epithelium in the IHC‐positive OLP samples in the present study. These vacuoles may demonstrate the features of liquefaction degeneration of basal cells. However, the nature of the vacuoles is unclear.

Inflammasomes are multiprotein complexes that are assembled by pattern recognition receptors following the detection of pathogenic microorganisms and danger signals in the cytosol of host cells; these complexes control inflammatory responses and coordinate antimicrobial host defense.^24^ It was recently demonstrated that *M. salivarium* activates inflammasomes, which induce the release of IL‐1β and pyroptosis (caspase‐1‐dependent inflammatory cell death), in murine dendritic cells and human monocytes.^25^ Although this result was obtained by in vitro research, the same phenomenon may occur in vivo in the subepithelial region of the lamina propria of the oral mucosa, where *M. salivarium* is present, because the tissues of the lamina propria contain dendritic cells and monocytes. Therefore, *M. salivarium* is suggested to play a role in activating inflammasomes in the lamina propria.

In conclusion, the present study demonstrated the existence of *M. salivarium* in the epithelium and lamina propria in approximately 60% of OLP samples. Furthermore, a close relation between the presence of mycoplasma and sawtooth rete ridge formation was demonstrated. However, the role of *M. salivarium* in the epithelium and lamina propria of OLP tissue could not be elucidated.

This study represents preliminary research to examine the presence of *M. salivarium* in OLP tissues by IHC. Credible data demonstrating the pathogenesis of OLP could not be obtained. In addition, this study has several limitations, including the small number of OLP samples. However, we believe that the findings obtained from this study will be useful to elucidate the etiological factors and pathogenesis of OLP in the future. Further studies are necessary to clarify the role of *M. salivarium* in OLP.

## CONFLICT OF INTEREST

The authors declare no conflict of interests associated with this manuscript.
